# Active protein aggregates induced by terminally attached self-assembling peptide ELK16 in *Escherichia coli*

**DOI:** 10.1186/1475-2859-10-9

**Published:** 2011-02-15

**Authors:** Wei Wu, Lei Xing, Bihong Zhou, Zhanglin Lin

**Affiliations:** 1 Department of Chemical Engineering, Tsinghua University, One Tsinghua Garden Road, Beijing 100084, PR China

## Abstract

**Background:**

In recent years, it has been gradually realized that bacterial inclusion bodies (IBs) could be biologically active. In particular, several proteins including green fluorescent protein, β-galactosidase, β-lactamase, alkaline phosphatase, D-amino acid oxidase, polyphosphate kinase 3, maltodextrin phosphorylase, and sialic acid aldolase have been successfully produced as active IBs when fused to an appropriate partner such as the foot-and-mouth disease virus capsid protein VP1, or the human β-amyloid peptide Aβ42(F19D). As active IBs may have many attractive advantages in enzyme production and industrial applications, it is of considerable interest to explore them further.

**Results:**

In this paper, we report that an ionic self-assembling peptide ELK16 (LELELKLK)_2 _was able to effectively induce the formation of cytoplasmic inclusion bodies in *Escherichia coli *(*E. coli*) when attached to the carboxyl termini of four model proteins including lipase A, amadoriase II, β-xylosidase, and green fluorescent protein. These aggregates had a general appearance similar to the usually reported cytoplasmic inclusion bodies (IBs) under transmission electron microscopy or fluorescence confocal microscopy. Except for lipase A-ELK16 fusion, the three other fusion protein aggregates retained comparable specific activities with the native counterparts. Conformational analyses by Fourier transform infrared spectroscopy revealed the existence of newly formed antiparallel beta-sheet structures in these ELK16 peptide-induced inclusion bodies, which is consistent with the reported assembly of the ELK16 peptide.

**Conclusions:**

This has been the first report where a terminally attached self-assembling β peptide ELK16 can promote the formation of active inclusion bodies or active protein aggregates in *E. coli*. It has the potential to render *E. coli *and other recombinant hosts more efficient as microbial cell factories for protein production. Our observation might also provide hints for protein aggregation-related diseases.

## Background

Bacteria have long served as cell factories for recombinant protein production, while higher yields of soluble proteins have always been pursued. However, over-expression of heterogenous proteins often leads to the accumulation of insoluble protein aggregates, commonly known as inclusion bodies (IBs). The accumulation of IBs has been one main obstacle of recombinant protein production, as they are often clusters of misfolded polypeptides, which are thus disfunctional and undesirable [1]. However, over the last several years, it is gradually realized that some IBs could be still biologically active, without regard to their insolubility [2-4]. Partially active IBs were first observed for β-galactosidase in 1989 [5], likely as a result of a small amount of correctly folded protein entrapped in inactive unfolded polypeptide molecules [6]. True active IBs, in which the majority of the polypeptides remain active, were first reported in early 1991 [7], and have gained more attention since 2005 [8]. In particular, when used as fusion protein partners, the foot-and-mouth disease virus capsid protein VP1, the human β-amyloid peptide Aβ42(F19D) [8], a maltose-binding protein mutant (MalE31) [9], and the cellulose-binding domain of *Clostridium cellulovorans *(CBD_clos_) [10-14] have been shown to induce the formation of active inclusion bodies in *Escherichia coli *(*E. coli*) with specific activities ranging from about 20% to levels comparable with the native proteins [8,10]. These new findings have enhanced the understanding of protein folding, and also led to several studies about using these active inclusion bodies for immobilized biocatalysis [15], bioassays [13], and biomaterials [16].

In our previous study to increase the affinity of *Bacillus subtilis *lipase A (LipA) toward hydrophobic surfaces, we genetically attached to LipA an amphipathic helical peptide 18A that is capable of self assembly in aqueous solution [17,18]. Unexpectedly, the resulting fusion protein formed aggregates after being expressed in *E. coli*. Subsequently, *Aspergillus fumigatus *amadoriase II (AMA), *Bacillus pumilus *β-xylosidase (XynB), as well as green fluorescent protein (GFP) were tested and similar active protein aggregates with high specific activities were found (Wu W, Xing L, Zhou B, Cai Z, Chen B, Lin Z: Assembly of active protein aggregates in vivo induced by terminally attached amphipathic peptide, submitted).

**Figure 1:**
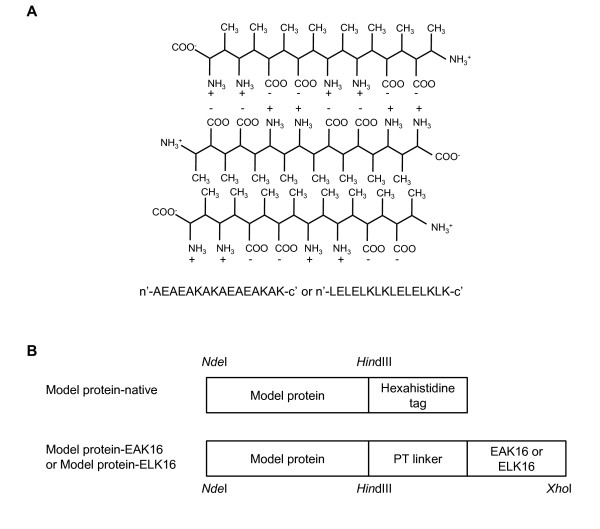
**Schematics for ELK16 aggregation and fusion protein constructs**. (A) Antiparallel β-sheet formed by the self-complementary EAK16 or ELK16 peptides. (B) Genetic constructs of the ELK16 fusion proteins. EAK16, (AEAEAKAK)_2_; ELK16, (LELELKLK)_2_; linker, PTPPTTPTPPTTPTPTP; model proteins, XynB, AMA, or GFP.

This serendipitous observation stimulated us to search for more peptides which could similarly induce the formation of active protein aggregates. A well-defined self-complementary amphipathic β peptide, EAK16 (AEAEAKAKAEAEAKAK, or (AEAEAKAK)_2_) [19], has attracted our attention. This peptide originates from the Zuotin protein sequence [20], and can spontaneously form β-sheet structure in aqueous solution with a proposed pattern shown in Figure 1A[19]. In this work, we wanted to investigate if EAK16 and one of its more hydrophobic variant, ELK16 (LELELKLKLELELKLK, or (LELELKLK)_2_), could function as an inducer for active protein aggregate formation. Once again, the peptides were genetically attached to the carboxyl termini of the model proteins LipA, AMA, XynB, as well as GFP, and the fusions were expressed in *E. coli *BL21 (DE3). The fusion proteins were characterized by enzymatic activity assays, transmission electron microscopy (TEM), laser scanning confocal microscopy (LSCM) and Fourier transform infrared spectroscopy (FTIR). The studies we have performed indeed show that the terminally attached ELK16 peptide, but not EAK16, can act as an effective inducer for the formation of active inclusion bodies *in vivo*.

## Results

### Active protein aggregates expressed in *E. coli *BL21 (DE3)

**Figure 2:**
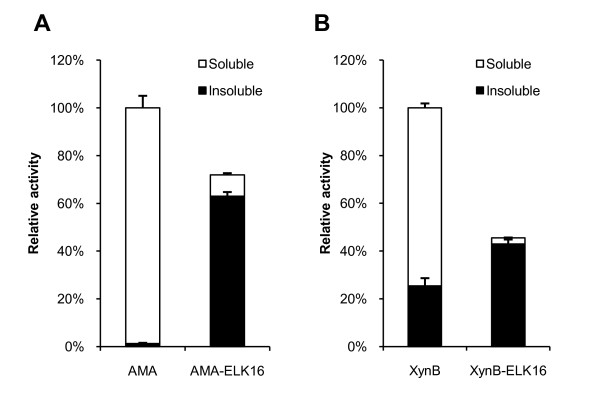
**Distributions of enzymatic activities in the soluble and insoluble fractions of cells lysates**. (A) AMA. (B) XynB. The activities were calculated using the average of three independent experiments and normalized to the total activities of the respective native enzyme extracted from a same amount of cells (OD_600_). Standard deviations are also showed.

In this work, the peptide EAK16 (AEAEAKAK)_2 _or ELK16 (LELELKLK)_2 _was terminally fused to the model proteins including LipA, AMA, XynB, and GFP (Figure 1A). Production of the fusion proteins was induced through the addition of isopropyl β-D-1-thiogalactopyranoside (IPTG). ELK16 fusion proteins were found to express as largely inclusion bodies, without disturbing the cell growth compared with the native proteins, as judged from the cell density measurements (OD_600_) (data not shown). On the other hand, all the EAK16 fusions were solubly expressed, with total activities and expression levels comparable to the native counterparts (data not shown), and thus were not further studied. Judging from the enzyme activities in the soluble and insoluble fractions (Figure 2), the aggregated fusion proteins accounted for 87.5% of the total activity for AMA-ELK16 and 94.4% for XynB-ELK16. Using the total activities of the two native enzymes as the respective benchmark, about 72% of AMA activity and 45% XynB activity could be obtained in the form of active inclusion bodies. Furthermore, both specific activities of the two fusion proteins were closed to the native enzymes (Table 1). XynB-ELK16 aggregates retained about 77% of specific activity compared with its native counterpart, while for AMA-ELK16 aggregates about 120%. On the other hand, it appears that for both AMA and XynB, the amount of ELK16 fusion produced was lower than the native counterpart. An unexpected result was that the LipA-ELK16 fusion was expressed in the form of almost totally inactive inclusion bodies. It is surmised that the addition of ELK16 might greatly influence the folding or catalytic site of this lipase [21], which does not contain a usual lid seen in many lipases and has a more exposed hydrophobic active site.

**Table 1:** Enzymatic activities of the fusion proteins produced in *E. coli*

	**Activity (U/ml)**^**1**^					
					
Enzymes	Soluble fraction	Insoluble fraction	**Percent of activity found in insoluble fraction**^**2**^	**Enzyme amount (mg/ml)**^**3**^	**Specific activity (U/mg enzyme)**^**3**^	**Specific activity relative to the native enzyme**^**3**^
AMA						
AMA-native	734.4 ± 37.5	9.63 ± 2.29	1.3%	0.47	1563	100%
AMA-ELK16	66.7 ± 5.1	468.7 ± 12.9	87.5%	0.25	1875	120%
XynB						
XynB-native	398.8 ± 9.7	136.2 ± 17.0	25.4%	0.31	1290	100%
XynB-ELK16	13.6 ± 0.6	230.0 ± 9.8	94.4%	0.23	991	77%

^1^Cells were collected 6 h after IPTG induction. 1 ml soluble enzyme was extracted from 10 OD_600 _of cells; the insoluble fraction was also from 10 OD_600 _of cells and then re-suspended in 1 ml of lysis buffer. Enzymes amounts were calculated based on SDS PAGE with serial concentrations of BSA as standards. AMA: *Aspergillus fumigatus *amadoriase II; XynB: *Bacillus pumilus *β-xylosidase.

^2^The percentage of the activity found in the insoluble fraction relative to the total activity of the native or ELK16 fusion protein in the cell lysate (soluble and insoluble fractions combined).

^3^For the native enzyme, the value concerns the enzyme in the soluble fraction; while for the ELK16 fusion, the value concerns the enzyme in the insoluble fraction (more specifically, enzyme aggregate).

**Figure 3:**
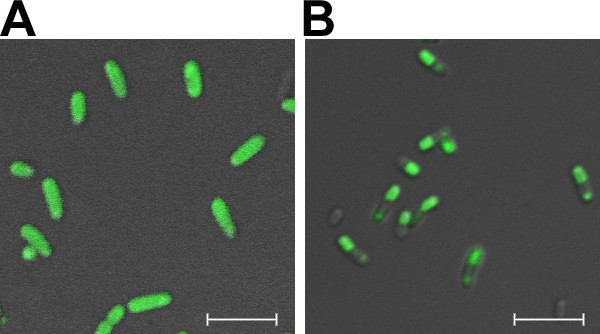
**Intracellular localization of GFP-ELK16 in *E. coli *BL21 (DE3) cells**. (A) GFP. (B) GFP-ELK16. The merged images of the confocal fluorescent micrographs and the differential interference contrast micrographs (DIC) are shown. Size bars, 5 μm.

**Figure 4:**
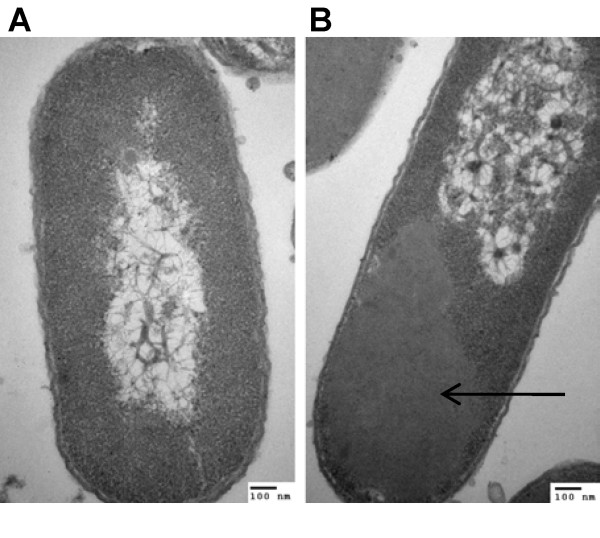
**Intracellular localization of the ELK16 fusion proteins in *E. coli *BL21 (DE3) cells**. (A) Native AMA. (B) AMA-ELK16. The arrow shows the newly formed inclusion body in the AMA-ELK16 cell. Size bars, 100 nm.

The distribution of the fusion protein was also analyzed by fluorescence confocal microscopy with GFP as the model protein, by TEM for AMA and XynB. As shown in Figure 3, while GFP-expressing cells exhibited uniform green fluorescence throughout the cytoplasm (Figure 3A), an obvious localized distribution of fluorescence could be observed from the GFP-ELK16 cells (Figure 3B). This observation clearly confirms the *in vivo *formation of the active inclusion bodies for GFP-ELK16, in which the GFP was fluorescent, and thus retained the active conformation. Moreover, the TEM image of the thin-sectioned cells expressing AMA-ELK16 (Figure 4B) clearly shows that a large portion of the cytoplasm was occupied by the newly formed inclusion bodies [22], which was however hardly observed in the native AMA cells (Figure 4A). A similar TEM image was observed for XynB-ELK16, and for GFP-ELK16 (data not shown).

### FTIR evidence for amyloid-like structure in the active inclusion bodies

**Figure 5:**
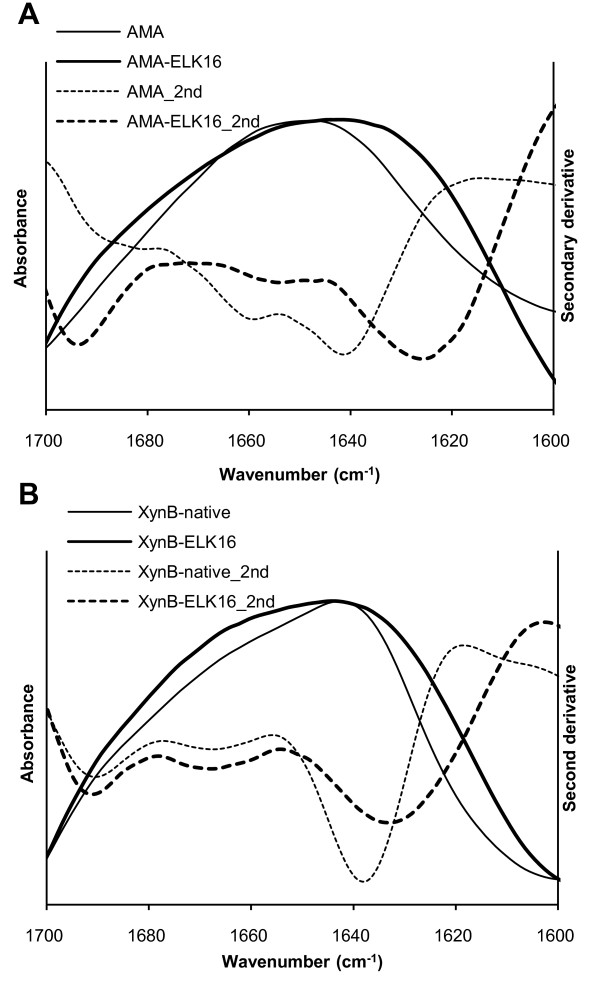
**FTIR spectra of ELK16 aggregates (thick lines) and the respective native proteins (thin lines)**. The amide region I between 1600 cm^-1 ^and 1700 cm^-1 ^for (A) AMA (B) XynB with or without ELK16 are shown. The spectra are smoothed and scaled independently to be full scale on the absorbance axis [25]. The second derivatives are also showed as dashed lines.

To confirm the existence of the hypothetic β-sheet (Figure 1A) [19] in the ELK16 aggregates, ELK16 fusions were analyzed by FTIR, one of the few methods available for dissecting the secondary structural information of protein aggregates [23,24]. Generally, the amide region I of FTIR absorbance (between 1600 cm^-1 ^and 1700 cm^-1^), as well as the secondary derivatives of the absorbances, have been used to assign the secondary structures [23]. For AMA (Figure 5A), the FTIR spectrum of AMA-ELK16 aggregates exhibits a maximum at 1643 cm^-1 ^(thick solid line), with a red shift of 4 cm^-1 ^compared with that of the native AMA (1647 cm^-1^) (thin solid line), indicating an increase in the β-sheet component for this α/β protein [25,26]. In terms of the secondary derivatives, peaks near 1615-1643 cm^-1^, 1647-1654 cm^-1^, and 1650-1660 cm^-1 ^are believed to be indicative of β-sheet, random coil, and α-helical conformations, respectively [23,27,28]. In particular, the presence of bands near 1621-1625 cm^-1 ^and 1694 cm^-1 ^is considered to arise from extended antiparallel pleated β-sheet structures [28,29]. For AMA-ELK16, new bands appearing at 1625 cm^-1 ^and 1693 cm^-1 ^in the secondary derivative in the ELK16 fusion (thick dashed line) are consistent with the formation of extended antiparallel pleated β-sheet structures in the AMA-ELK16 aggregate. For XynB (Figure 5B), the difference between the FTIR spectra of the native XynB and XynB-ELK16 is less distinctive. It is probably because the native XynB is an all-β protein, with estimated 46% beta structure based on its homology to *B. subtilis *XynB (PDB code: 1YIF) (Patskovsky and Almo, unpublished data), thus the difference is more subtle [23]. Nonetheless, the shift trend is similar. The spectra of both XynB-ELK16 and XynB exhibit a maximum at about 1643 cm^-1^, while the former is broader. However, the secondary derivative of XynB-ELK16 exhibits a dominant band at 1633 cm^-1 ^and a less dominant band at 1691 cm^-1^, while that of the native XynB shows bands at 1637 cm^-1 ^and 1691 cm^-1^.

## Discussion

Our results clearly demonstrate that the terminally-fused peptide ELK16 can induce the formation of active protein aggregates. Those aggregates are morphologically similar with previously well-studied inclusion bodies in *E. coli *[22], however, proteins accumulated in these ELK16-induced inclusion bodies still largely retain their activity. It has been reported that the assembly of the EAK16 peptide (e.g., EAK16, ELK16, RAD16) is mainly influenced by two factors, the ionic complementary interactions between the negatively charged Glu or Asp and the positively charged Lys or Arg, and the extent of the hydrophobic residues [19,30]. While EAK16 alone can spontaneously assemble in aqueous solution, in our experiment it failed to induce the aggregation of target proteins. By replacing Ala in EAK16 with the more hydrophobic residue Leu, the resulting ELK16 peptide successfully induced the *in vivo *assembly of active inclusion bodies, in which extended antiparallel pleated β-sheet structures were revealed by the FTIR analyses. It is thus surmised that hydrophobic interactions in the ELK16 peptide play an important role in inducing the formation of active protein aggregates.

Since the proteins in the aggregates are active, it implies that the secondary and tertiary structures of the proteins must be largely preserved. Given the well-established mechanism of the self-assembly of the EAK16 peptide and alikes [30], as well as the existence of amyloid-like fibrils in the Aβ42 induced active inclusion bodies [31], we can reasonably conclude that the model proteins (AMA, XynB, and GFP) fold independently of the ELK16 moiety of the fusions; the folded model proteins however are aggregation-prone in the cytoplasmic aqueous environment due to the attached self-assembling ELK16 peptide. In other words, the model protein "moieties" are "immobilized" by the ELK16 moiety.

Compared with the previous reported aggregation-prone fusion partners for active inclusion bodies formation, the peptide ELK16 used in this work is much shorter in size, with simpler secondary structure, and easy to modulate. The active protein aggregates formed by the ELK16 fusion proteins have attractive application potentials in biotechnology, owing to their insolubility but high activity. For example, the ELK16 peptide-induced active aggregation method could provide an effective way for *in-situ *carrier-free enzyme immobilization and reduce the cost in otherwise necessary enzyme concentration, purification, and chemical immobilization steps [15], with less activity loss. The insolubility of the ELK16 fusion proteins might also provide a possibility for use in the production of toxic peptides or proteins, for which larger fusion partners were already attempted [32-34]. Moreover, it is possible to develop a quick scheme for protein expression and purification in bacteria, by adding an appropriate cleavable site [35] between the target protein and the ELK16 peptide, whose feasibility has already been proved in our ongoing preliminary work. We also surmise that our observation on this peptide-induced aggregation might provide insight for the understanding of protein folding, misfolding and aggregation, as well as for protein aggregation-related diseases [24,36].

## Conclusions

In conclusion, the self-assembling peptide ELK16 has been employed for the first time for the production of active inclusion bodies in *E. coli*, which is mostly likely induced via the intermolecular beta structure formed by ELK16. Due to the simplicity of this peptide and the high efficiency of the peptide-induced aggregation, it is of great interest to explore this approach further for applications in protein expression, purification, and industrial biocatalysis.

## Methods

### Plasmid Construction

The plasmid pET30a (+) (Novagen) was modified to generate corresponding plasmids encoding fusions of target proteins, PT type linker, and EXK16 (X = A or L). The primers LipA-For (5'ACGACGACATATGGCTGAACACAATCCAGT 3', the restriction site is underlined.) and EAK-Rev (5' TCGTTCTCGAGTCATTTAGCCTTGGCCTCAGCTTCCGCTTTCGCCTTCGCTTCTGCTTCAGCCGGCGTCGGGGTTGGGGTGGTTGG 3', for EAK16) or ELK-Rev (5' TCGTTCTCGAGTCATTTCAGCTTTAATTCTAATTCCAGTTTTAACTTCAGTTCAAGTTCCAGCGGCGTCGGGGTTGGGGTGGTTGG 3', for ELK16) were used to amplify the gene encoding LipA-EXK16 (X = A or L) from the previously constructed pAc18A-LipA (Wu W, Xing L, Zhou B, Cai Z, Chen B, Lin Z: Assembly of active protein aggregates in vivo induced by terminally attached amphipathic peptide, submitted). The amplified LipA-EXK16 (X = A or L) genes were then restriction digested with *Nde*I and *Xho*I, and subcloned into the pET30a (+) vector to obtain the pET30a-LipA-EXK16 (X = A or L) constructs.

Other constructs, including pET30a-AMA-EXK16 (X = A or L) (AMA-For: 5' TTCTGGACATATGGCGGTAACCAAGTCATC 3' and EAK-Rev or ELK-Rev, respectively), pET30-XynB-EXK16 (X = A or L) (primers: XynB-For 5' ATGAGCACATATGAAGATTATCAATCCAGTGCTC 3', and EAK-Rev or ELK-Rev, respectively), and pET30-GFP-EXK16 (X = A or L) (primers: GFP-For 5' TTCTGGACATATGAGTAAAGGAGAAGAACTTT 3', and EAK-Rev or ELK-Rev, respectively) were similarly constructed (Figure 1B).

Fusion genes encoding the native proteins with the hexahistidine tag at the C-termini were also similarly amplified from the plasmids mentioned above with the former primers and reverse primers (LipA-Rev: 5' AAATTTAAGCTTATTCGTATTCTGGCCCCCGC-3', AMA-Rev: 5' GGGAGGAAGCTTTAACTTGGAAATATCTCTATAT 3', XynB-Rev: 5' GAAGAAGCTTTTCGTCTGTTTCCTCATAACG 3', and GFP-Rev: 5' AAGAGAAAGCTTATTCAGCTTGGCTGCAGGTCGAC 3', respectively), and subcloned into the pET30a (+) vector.

### Expression and purification of IBs

*E. coli *BL21 (DE3) cells were employed to express the fusions of AMA-ELK16 and XynB-ELK16, respectively. The expression conditions were generally similar as in the previous 18A work (Wu W, Xing L, Zhou B, Cai Z, Chen B, Lin Z: Assembly of active protein aggregates in vivo induced by terminally attached amphipathic peptide, submitted). In detail, the cell growth was carried out in Luria-Bertani medium supplemented with 50 mg/L kanamycin at 37°C with shaking (250 rpm). At an OD_600 _of 0.4-0.6, 0.2 mM isopropyl β-D-1-thiogalactopyranoside (IPTG) was added to initiate the protein expression at 30°C for another 6 h. Cells were harvested by centrifugation at 6,000 × g for 10 min and resuspended in lysis buffer (50 mM Tris-HCl, 50 mM NaCl, 5% glycerol, pH 7.2), followed by sonication on ice to lyse the cells thoroughly. Centrifugation (15,000 × g for 15 min) was preformed to separate the insoluble protein aggregates (IBs) from the clarified soluble fractions at 4°C. IBs were washed once with the same lysis buffer containing 0.5% (*v/v*) Triton X-100 and once with lysis buffer, and finally resuspended in the same volume of lysis buffer. The amounts of target proteins in both fractions were determined densitometrically by denaturing polyacrylamide gel electrophoresis (SDS PAGE) using bovine serum albumin (BSA) as standard [6].

### Determination of enzymatic activities in IBs

The activities of target proteins in both the soluble and insoluble fractions were measured in 96-well microplates with a SPECTRAMAX M2 microtiter reader (Molecular Device, CA). In detail, the amadoriase activity was measured at 37°C by monitoring the formation of a quinone dye following A_555 _(ε, 39.2 cm^2^/μmol) in a peroxidase-coupling reaction [37]. The reaction mixture contained 100 mM potassium phosphate buffer (pH 8.0), 2.7 purpurogallin units of peroxidase, 0.45 mM 4-aminoantipyrine, 0.5 mM *N*-ethyl-*N*-(2-hydroxy-3-sulfopropyl)-*m*-toluidine (TOOS), and 5.0 mM D-fructosyl-glycine in a total volume of 180 μl. The β-xylosidase and lipase assays were carried out at 37°C by monitoring the formation of *p*-nitrophenol (pNP) following A_405 _(ε, 18.7 cm^2^/μmol). The β-xylosidase reaction [38] was performed in 180 μl reaction system (50 mM phosphate buffer, pH 6.0, 2.5 mM *p*-nitrophenyl β-D-xylopyranoside). The lipase assay [39] was performed by addition of 5 μl diluted enzyme into 175 μl reaction buffer (50 mM sodium phosphate buffer, pH 8.0, 0.4 mM *p*-nitrophenyl palmitate, 0.2% sodium deoxycholate, 0.1% gum arabic). One unit of enzyme activity was defined as the amount of enzyme that produced 1 nmol H_2_O_2 _or *p*-nitrophenol per min.

### Laser scanning confocal microscopic (LSCM) analyses

The cells expressing unmodified GFP or GFP-ELK16 were cultivated at 23°C for 22 h after 0.2 mM IPTG induction. Then the cells were harvested, fixed with 4% paraformaldehyde and photographed at 488 nm using a Zeiss LSM 710 confocal microscope (Carl Zeiss, Germany).

### Transmission electron microscopic analyses of cells producing ELK16 fusion proteins

Morphometric analyses of the aggregates were also performed with a Hitachi H-7650B (Hitachi, Japan) transmission electron microscope follow the similar sample processing protocol as previously described (Wu W, Xing L, Zhou B, Cai Z, Chen B, Lin Z: Assembly of active protein aggregates in vivo induced by terminally attached amphipathic peptide, submitted). In detail, the cells were collected after induction of protein expression. 2.5% glutaraldehyde and 2% osmium tetraoxide were used successively to fix the cells. After a graded-ethanol serial dehydration step, the cells were embedded in epoxy resins, sectioned, stained by uranyl acetate solution and lead citrate, and then observed with a Hitachi H-7650B (Hitachi, Japan) transmission electron microscope at an accelerating voltage of 80 kV.

### Conformational analyses by Fourier transform infrared spectroscopy (FTIR)

For FTIR spectroscopy analysis, the cells containing AMA-ELK16 or XynB-ELK16 were lysed following the lysozyme-based protocol as described previously (Wu W, Xing L, Zhou B, Cai Z, Chen B, Lin Z: Assembly of active protein aggregates in vivo induced by terminally attached amphipathic peptide, submitted). Cell pellets were resuspended using the lysis buffer, followed by 1 mg/ml lysozyme treatment and DNaseI treatment in 37°C for 1 h to lyse the cells. The IBs were then separated by centrifugation, and washed stepwise by sterile PBS buffer containing 0.5% (*v/v*) Triton X-100 and by PBS buffer, and dried for 12 h in a Freeze-Vac system. The FTIR absorption spectra were acquired in transmission from 4000 to 650 cm^-1 ^by a Nicolet Nexus 670 FTIR Spectrometer with a nitrogen-cooled, mercury - cadmium - tellurium (MCT) detector. For each spectrum, 50 interferograms were collected and averaged with 4 cm^-1 ^spectral resolution. The amide region I of the IR spectra (between 1600 cm^-1 ^and 1700 cm^-1^) were smoothed by 13 points, and scaled to be full scale on the absorbance axis. The second derivatives of the measured absorption spectra were calculated by the Savitzky-Golay method (3rd polynomial, 13 smoothing points) using the OMNIC 8.0 software (Thermo Fisher Scientific Inc.).

## Competing interests

The authors declare that they have no competing interests.

## Authors' contributions

WW designed part of the experiments, performed most of the experiments, and prepared the manuscript draft. LX and BZ participated in the enzymatic assays and instrumental analyses. ZL conceived the study, designed and supervised the experiments, and revised the manuscript. All authors read and approved the final manuscript.
